# Bone Remodeling Around Implants with Different Macro-Design Placed in Post-Extraction Sockets: A Cone-Beam Computed Tomography (CBCT) Randomized Controlled Clinical Trial (RCT)

**DOI:** 10.3390/dj13020078

**Published:** 2025-02-11

**Authors:** Roberta Grassi, Fábio França Vieira e Silva, Gennaro Musella, Francesco Pettini, Gisela Cristina Vianna Camolesi, Martina Coppini, Stefania Cantore

**Affiliations:** 1 Higher Education Institution, Malta ICOM Educational, GZR 1075 Il-Gzira, Malta; 2 Oral Medicine, Oral Surgery and Implantology Unit (MedOralRes), Faculty of Medicine and Dentistry, University of Santiago de Compostela, San Francisco Street, s/n, 15782 Santiago de Compostela, Spain; fabio.franca@unicampania.it (F.F.V.e.S.); giselacristina.vianna@rai.usc.es (G.C.V.C.); 3 Health Research Institute of Santiago de Compostela (FIDIS), ORALRES Group, Santiago de Compostela University Clinical Hospital, University of Santiago de Compostela, Choupana Street, s/n, 15706 Santiago de Compostela, Spain; 4 Department of Precision Medicine, University of Campania Luigi Vanvitelli, Via L. De Crecchio, 7, 80138 Naples, Italy; 5 Department of Clinical and Experimental Medicine, University of Foggia, 71100 Foggia, Italy; 6 Department of Interdisciplinary Medicine, University of Bari Aldo Moro, Via Piazza G, Cesare, 11, 70123 Bari, Italy; francesco.pettini@uniba.it; 7 Unit of Oral Medicine and Dentistry for Frail Patients, Department of Rehabilitation, Fragility and Continuity of Care, University Hospital Palermo, 90134 Palermo, Italy; martina.coppini@unipa.it; 8 Regional Dental Community Service “Sorriso& Benessere—Ricerca e Clinica”, Azienda Sanitaria Locale of Bari-ASL/BA, 70129 Bari, Italy

**Keywords:** clinical research, CT imaging, implant macro-design, radiology, patient-centered outcomes, bone–implant interactions

## Abstract

**Background:** Immediate post-extraction dental implants are increasingly popular, but ensuring primary stability and managing peri-implant tissues remain challenging. Implant macro-design significantly impacts stability and osseointegration. This study used Cone-beam Computed Tomography (CBCT) to evaluate changes in alveolar bone following immediate placement of two implant designs, System 2P and Dura-Vit 3P, which feature semi-conical microgeometry and apical self-tapping portions for improved stability and bone regeneration. **Methods:** With a 1:1 allocation ratio, the current investigation was a two-arm parallel group randomized clinical trial. Patients qualified if they required immediate dental replacements with adequate buccal bone support. Two types of implants were placed: System 2P (cylindrical shape) and Dura-Vit 3P (more conical shape, with a particular architecture of threads). Following the intervention, CBCT was performed both immediately (T1) and six months later (T2). Measurements of CBCT horizontal bone level at apical, medial, and bevel height on the palatal/lingual and vestibular sides as well as the buccal vertical gap were the primary results. Complications, implant stability quotient (ISQ), and torque insertion were evaluated. The Mann–Whitney test was used to determine time-based differences within each group, while the Wilcoxon test was used to estimate differences between groups. The impact of baseline marginal gap dimension and gingival biotype was estimated using multiple regressions. **Results:** Thirty patients were recruited and randomized to treatments, with two lost to follow-up. One System 2P implant failed and two patients of the Dura-Vit 3P group dropped out. At T1, the Dura-Vit 3P group exhibited a lower mean insertion torque and a higher ISQ than the System 2P group. Furthermore, the Dura-Vit 3P group showed lower bone reduction compared to System 2P at horizontal and vertical measurements with significant differences for the vestibular and palatal base and medial level (*p*-values < 0.05). Regression models indicated a positive effect of thick biotypes on gap filling and dimensional bone reduction. No complications were observed in both groups. **Conclusions:** The Dura-Vit 3P implant exhibits high primary stability when inserted in post-extraction sites. Furthermore, this kind of implant stimulates higher bone stability on both the palatal and buccal side when compared to the System 2P implant. The present findings support the evidence that the macro-design of the Dura-Vit 3P implant promotes increased primary stability and reduces bone loss.

## 1. Introduction

Implant-supported rehabilitation is a cost-effective and durable solution for replacing missing teeth, offering both functional and esthetic benefits [1]. Dental implants placed in immediate post-extraction sites represent an increasing trend in implant dentistry. The high survival and success rate as well as the a reduced number of interventions, shorter comprehensive treatment time, and potential immediate prosthesis are important aspects that encourage clinicians to choose immediate implants over delayed ones whenever possible [2,3,4]. Beyond functional and esthetic benefits, maintaining dentition through immediate implant placement may also have broader health implications. Recent evidence links tooth loss in periodontitis patients to mild cognitive impairment, likely due to systemic inflammation and reduced mastication [5], highlighting the importance of timely implant rehabilitation for overall health.

The inherent post-extraction implantation challenges consist of ensuring primary stability and managing peri-implant tissues to achieve successful osseointegration and optimal healing. Preserving the health of these tissues is not only essential for implant success but may also play a role in mitigating systemic effects linked to oral disease, reinforcing the broader significance of maintaining a healthy dentition [6]. Many studies have widely addressed the factors influencing implant success and have been reported by numerous authors, testing approaches such as bone grafting in the buccal jump space or buccal bone plate preservation. However, different studies report controversial results on the benefits of bone grafting [7,8,9], while the preservation of the buccal bone plate has been proven to be a major factor for the immediate success of implants [10,11].

Furthermore, soft tissue and crestal bone changes around implants remain critical considerations, as they can significantly influence long-term outcomes [12]. The use of platelet-rich plasma (PRP) and platelet-rich fibrin (PRF) has also emerged as a promising approach for accelerating bone healing, particularly in the post-extraction phase [13,14]. Advanced surface modifications, such as chitosan-based coatings, offer potential to further optimize implant systems by enhancing osseointegration and providing antibacterial protection, thereby improving long-term success rates [15].

The primary stability remains the most crucial aspect to consider to ensure a successful osseointegration with post-extraction implants. While surgical site preparation generally impacts primary stability [16], implant macro-design is widely recognized as a key determinant [17,18,19]. Recent scientific evidence suggests that various isolated macro-design characteristics such as thread type, thread pitch, thread depth, and face angle should be studied so that the implant geometry can balance the compressive stress and tensile stress and produce a minimum shear force [17,20]. Studies have reported that semi-conical microgeometry with an apical self-tapping portion results in higher initial stability even in the presence of buccal bone defects and exhibits better buccal bone regeneration and increased bone-to-implant contact [17,21].

The present study aims to estimate primary stability and bone response around a novel conical implant design (Dura-Vit 3P, B&B Dental Implant Company, Bologna, Italy) for immediate implantation as compared to same-manufacturer standard cylindrical/low angle conical “V”-shape design (System 2P, Bone System, Milan, Italy). Both the implants were designed to be placed at bone/tissue level, but with some differences in terms of geometry design and surface treatment. A typical feature of System 2P is the transmucos collar, and it presents a micro-structured surface with a high level of decontamination (fine grain sandblasting), with “double attack” acidification and Argon Plasma treatment to reduce healing times. The Dura-Vit 3P implant exhibits a more conical shape and presents a self-tapping system, which aim to provide greater primary stability. Furthermore, there are large inter-thread dimensions (triple-threated spirals) and wide spaces created between the margin of the threads and the body of the implant that might serve as healing cambers for the blood and the drilled bone surrounding the implant, therefore resulting in an improved bone reaction, also related to the surface treatment for implants (grit blasted and acid etched surface topography).

This randomized clinical trial (RCT) aimed to assess primary stability and evaluate dimensional alterations in the buccal and palatal socket walls, as well as the healing of marginal defects, following the immediate post-extraction placement of Dura-Vit 3P and System 2P implants, using Cone-beam Computed Tomography (CBCT) measurements. The null hypothesis was that Dura-Vit 3P implants would result in better primary stability and reduced surrounding bone loss as compared to System 2P implants.

## 2. Methodology

### 2.1. Study Design

This study is a two-arm parallel-group randomized clinical trial with a 1:1 allocation ratio. All participants received comprehensive information about the treatment procedure and provided informed consent for study participation, therapy, and adherence to follow-up protocols. The interventions were conducted in accordance with the ethical principles outlined in the revised Helsinki Declaration for biomedical research involving human subjects [22]. The study protocol and informed consent forms received approval from the Local Ethical Committee of Regione Puglia Azienda Bari-ASL/BA (registration number 0155) on 27 January 2021. The study was conducted and reported following the CONSORT statement [23] and in compliance with the EQUATOR guidelines. This clinical trial was registered at Clinicaltrials.gov (ID: NCT04492111, register date 2 September 2020).

### 2.2. Randomization and Blinding

Each of the three operators placed 10 implants, totaling 15 Dura-Vit 3P (B&B Dental Implant Company, Bologna, Italy) and 15 System 2P (Bone System, Milan, Italy) implants, with each patient receiving a single implant. Randomization was achieved through a block randomization list with variable block sizes of 3 and 6, generated using the “ralloc” command in Stata (StataCorp, College Station, TX, USA). Treatment allocation was centralized and disclosed to the surgeon only after tooth extraction and socket debridement. Operator blinding was not feasible; however, both patients and primary outcome assessors remained blinded. A single independent investigator (SC), experienced in radiological measurements, conducted all CBCT assessments using anonymized data. Statistical analyses were performed by an independent clinician (FFSV) with expertise in statistical analysis, utilizing a de-identified database without references to specific groups or patients.

### 2.3. Study Population

Adult patients with the clinical indication of immediate implant placement were assessed for enrolment. All patients were enrolled and treated at the ASL Bari by 3 different experienced surgeons (R.G., G.M. and S.C). The study applied the following selection criteria:

1. No systemic diseases that could interfere with implant integration or healing.
2. Satisfactory periodontal health, defined as a Modified Sulcus Bleeding Index below 30% and a Russell’s Periodontal Index below 2.
3. No active or untreated chronic periodontal disease.
4. Presence of adjacent natural teeth.

Additional conditions specific to post-extraction implantation included the following:

1. At least 4 mm of apical bone to ensure primary stability, assessed via periapical radiographs.
2. A buccal bone plate loss not exceeding 3 mm, determined by clinical sounding.
3. No chronic periapical lesions resulting in significant bone loss.
4. A minimum of 1 mm of keratinized marginal gingiva.

Individuals who had undergone CT scanning in the past five years or were heavy smokers (more than 10 cigarettes per day) were excluded from the study.

### 2.4. Surgical Procedures

**Figure 1:**
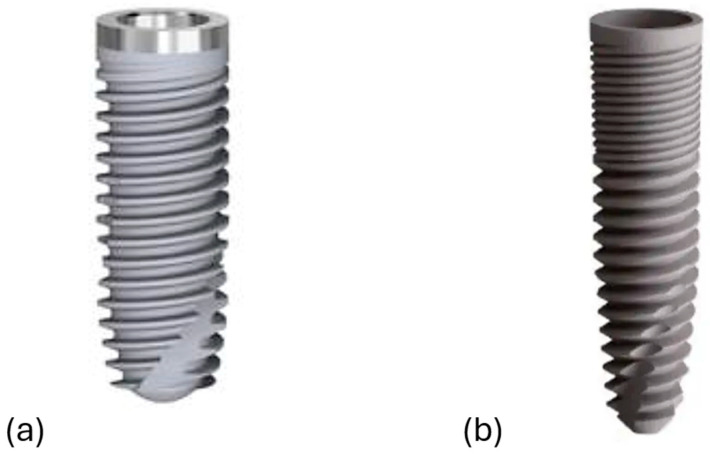
The dental implants’ macro-design. (**a**) System 2P and (**b**) Dura-Vit 3P.

All patients meeting the inclusion criteria were enrolled and treated at the Azienda Sanitaria Locale of Bari. The initial clinical evaluation included periapical radiographs and a comprehensive assessment of periodontal indices. Prior to surgery, all patients underwent a session of oral hygiene debridement. To minimize the risk of postoperative infections, prophylactic antibiotic therapy (600 mg clindamycin) was administered two hours before the procedure [24,25]. Surgical sites were anesthetized using computer-assisted local injections of articaine with adrenaline (1:100,000). Tooth extractions were performed carefully with luxators and forceps, avoiding any traumatic movements to preserve as much buccal alveolar bone as possible. In cases of periodontal inflammation, the alveolus was debrided to remove inflamed tissue. After this step, an assistant opened a sealed envelope containing the assigned implant type and informed the surgeon of the corresponding surgical procedure. The macro-design of both implant types is illustrated in Figure 1.

Implant dimensions were to be decided based on the anatomy of the post-extraction site and the required primary stability. The available diameters for the System 2P implant (Bone System, Milan, Italy) are 3.5 mm, 4.1 mm, and 5.0 mm, whereas for the Dura-Vit 3P implant (B&B Dental Implant Company, Bologna, Italy) they are 3.5 mm, 4.0 mm, 4.5 mm, and 5.0 mm. The length of the System 2P implant is 8.20 mm, 10 mm, 12 mm, 13.5 mm, and 15.0 mm, while the length of the Dura-Vit 3P implant is 6.0 mm, 8.0 mm, 10 mm, 12 mm, and 14 mm.

**Figure 2:**
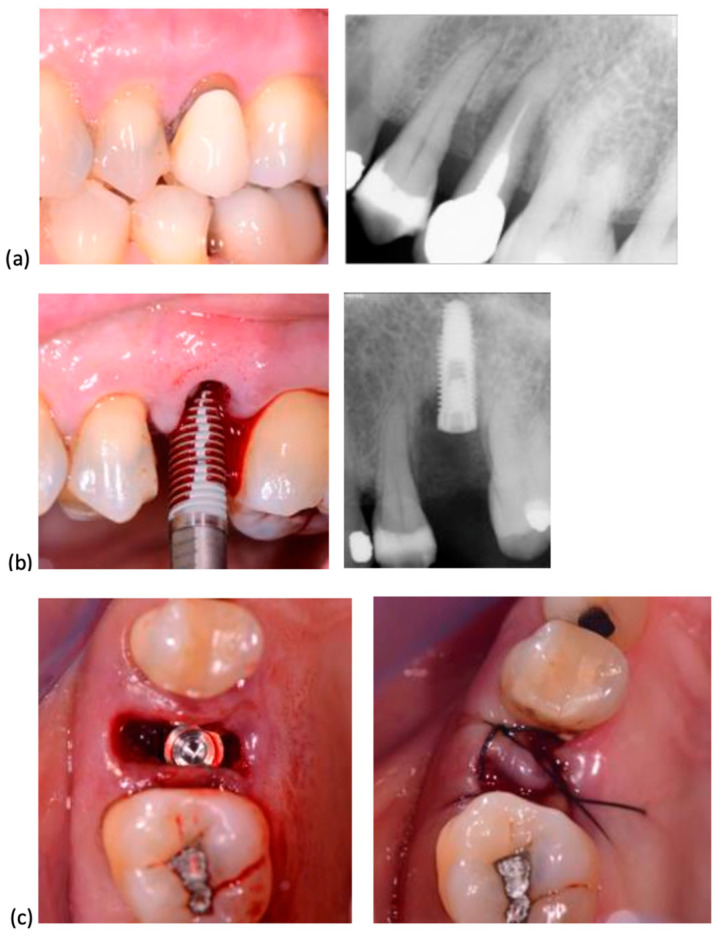
System 2P implant placement. (**a**) The second premolar, due to a vertical fracture, was extracted. (**b**) A System 2P implant was inserted without flap elevation. (**c**) The wound was closed with a single suture, without grafting.

**Figure 3:**
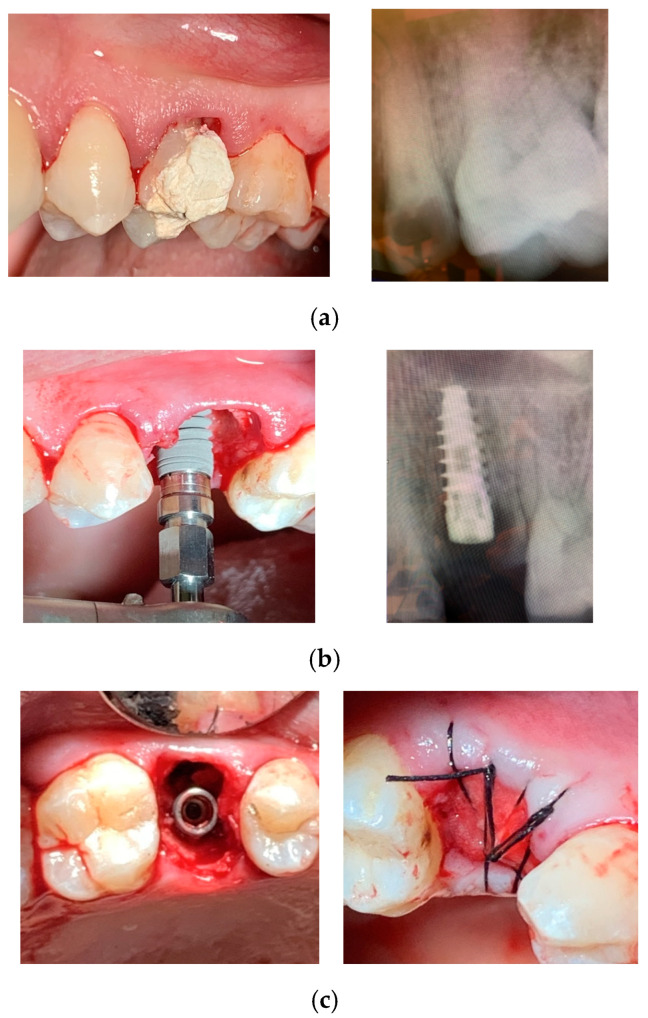
Dura-Vit 3P implant placement. (**a**) The first premolar was extracted due to a vertical fracture. (**b**) The implant was inserted without flap elevation. (**c**) The wound was closed with a single suture, without grafting.

In all cases, surgery was performed without elevating a flap. Following the established protocol, implant sites were prepared using progressively larger diameter twist drills, extending 2–3 mm beyond the alveolar site’s anatomical length to achieve primary stability. Implant placement began with a low-speed handpiece (20–25 rotations per minute) and was completed manually, applying a torque of 30–50 Ncm. The insertion torque was recorded using a surgical motor with a graphical interface displaying the maximum torque value. Implant shoulders were positioned 1 mm apically to the marginal level of the palatal bone. No grafting was performed in the residual space between the implant surface and socket walls. All implants were secured with cover screws, and primary closure was achieved through soft tissue mobilization and suturing. When necessary, a hemostatic collagen sponge (Septodont, Saint-Maur-des-Fossés, France) was placed beneath the suture. The insertion procedures for System 2P and Dura-Vit 3P implants, along with clinical and radiographic assessments for representative cases, are illustrated in Figure 2 and Figure 3, respectively.

Patients were instructed to chew on gauze soaked in tranexamic acid for a few minutes to promote blood clot formation. They received post-operative guidelines, including anti-inflammatory therapy if necessary and oral hygiene recommendations. A follow-up visit was scheduled 7 days after surgery for suture removal if required. Subsequent check-ups were conducted at 3 months and 6 months, the latter for the placement of the healing screw. Impressions for the final restoration were taken after 3 weeks, with full zirconia crowns delivered within 1–2 weeks.

### 2.5. Outcomes

**Figure 4:**
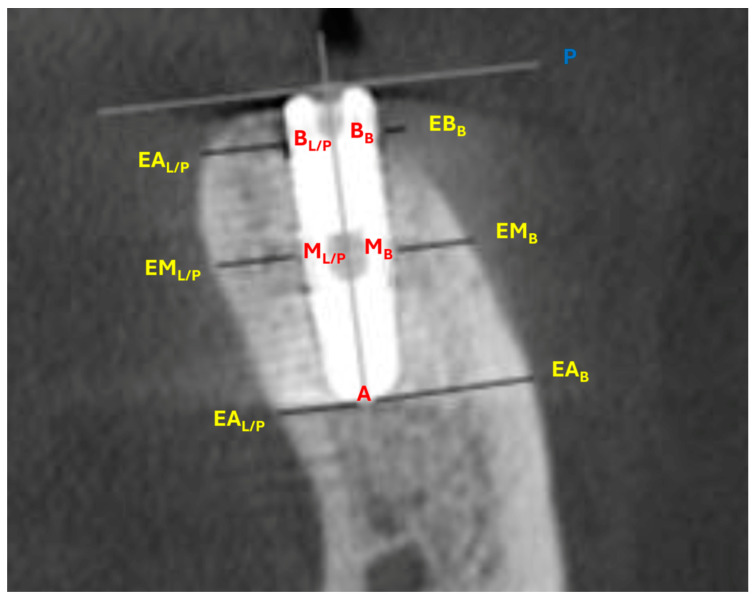
**CBCT landmarks and measurements.** Measurements were taken perpendicular to the implant vertical axis at the implant bevel plane. **Palatal/lingual apex**: distance from the most apical spire to the corresponding palatal/lingual external cortical bone landmark (A–EA_L/P_). **Palatal/lingual medial**: distance from the medial spire to the corresponding palatal/lingual cortical external bone landmark (M_L/P_–EM_L/P_). **Palatal/lingual base**: distance from the first spire to the corresponding palatal/lingual external cortical bone landmark (B_L/P_–EB_L/P_). **Vestibular apex**: distance from the most apical spire to the corresponding external buccal bone landmark (A–EA_B_). **Vestibular medial**: distance from the medial spire to the corresponding external buccal bone landmark (M_B_–EM_B_). **Vestibular base**: distance from the first spire to the corresponding external buccal bone landmark (B_B_–EB_L/P_). **Vestibular gap**: distance from the internal buccal bone landmark of the bevel/marginal spire to implant bevel plane (B_B_–P).

Demographic data and medical history, including age, sex, smoking habits, diabetes status, and periodontal disease history, were recorded. The gingival biotype was evaluated using Colorvue Biotype Probes (HU-FRIEDY, Milan, Italy) and categorized as thick or thin. The assessment involved inserting a white probe into the gingival sulcus; if visible through the tissue, the biotype was classified as thin. If not, a green probe was used, with visibility indicating medium thickness. If the green probe was not visible, the blue probe was used to classify the biotype as thick. For this study, the classification was simplified into two categories (thin or thick) by reassigning medium cases (green probe visible) to one of these groups to optimize data analysis and ensure clinical relevance. Bone dimensional changes around the implants were assessed using two consecutive CBCT scans (Gendex GXCB-500, Kavo, Biberach, Germany). The first scan (T1) was taken immediately after surgery, and the second (T2) was performed after six months of healing, during the appointment for healing screw placement. All CBCT scans were conducted at the same radiology center using consistent parameters (focal spot 0.5, FOV 5.0 × 5.0, voxel size 0.3, scan time 8.9 s, and 14-bit grayscale). The DICOM data were analyzed using i-CAT Vision™ software (ver. 1.8), which was used to minimize scattering artifacts and optimize image quality. CBCT measurements followed a standardized protocol [2,7], to assess buccal bone plate changes between the two time points. Horizontal measurements were taken perpendicular to the medial vertical axial section of the implant and parallel to the implant bevel plane, while the vertical vestibular gap was measured parallel to the medial vertical axis and perpendicular to the implant bevel plane. Figure 4 reports the CBCT landmarks and the measured linear outcomes:

- Three landmarks on the buccal and three landmarks on the lingual/palatal surface of the implant: most apical spire (AB for buccal surface and AL/P for lingual/palatal surface), medial spire (MB for buccal surface and ML/P for lingual/palatal surface), and bevel/marginal spire (BB for buccal surface and BL/P for lingual/palatal surface).
- Three corresponding external buccal and three corresponding external lingual/palatal bone landmarks determined perpendicular to the vertical axis of the implant (EAB for buccal bone and EAL/P for lingual/palatal bone, EMB for buccal bone and EML/P for lingual/palatal bone, EBB for buccal bone and EBL/P for lingual/palatal bone).
- The implant bevel plane (P).

CBCT measurements were taken twice by the same examiner (SC) at two different time points. The intraobserver error was assessed using the intraclass correlation coefficient and mean error to evaluate the repeatability of the measurements. Implant stability quotient (ISQ) values were recorded immediately after implant placement and again after six months using the Osstell Mentor device (Integration Diagnostic Ltd., Gothenburg, Sweden) with a SmartPeg Bone System. The SmartPeg sensor was screwed in and torqued to 10 Ncm, following the recommendations of the manufacturer. Measurements were taken in two directions (proximo-distal and anteroposterior), with the mean value calculated for each implant. Any intraoperative or postoperative complications were documented in detail at the time they occurred.

### 2.6. Sample Size

Since each patient received a single implant, the statistical unit of analysis was the patient. The required sample size was determined using a two-tailed alpha of 0.05 and a power of 0.9, assuming a potential standard deviation of 0.6 mm, as previously reported. The expected clinically significant difference between groups was set at 1 mm of bone at the marginal level. Accounting for a 20% potential loss to follow-up, the final sample size required was 15 participants per group.

### 2.7. Statistical Analysis

All data analysis was carried out following a pre-established analysis plan. Descriptive statistics were computed as means and standard deviation or proportion to report the included patients’ demographic profile and clinical characteristics, sites, and implants. The standard deviations of the within- and between-group mean differences were computed for each outcome, and the percentage changes in bone dimensions were also given. The Shapiro–Wilk test was used to verify the normalcy assumptions. The Mann–Whitney test was used to determine temporal differences within each group, while the Wilcoxon test was used to estimate differences between groups in terms of bone level and ISQ. Multiple linear regressions that adjusted for various potential predictive variables were used to examine the effects of smoking, soft-tissue biotype, and the initial dimension of the marginal gap on bone level alteration. The impact of diameter and length on the ISQ and crestal bone differences was also ascertained using regressions.

Analysis was conducted according to the intention to treat principle. The influence of data missing not at random had to be examined and bias quantified. It was necessary to analyze the impact of missing non-random data and quantify bias. Every statistical comparison was carried out at the 0.05 level of significance, and all tests were two-tailed. An operator who was blind used Stata version 13 (Stata Statistical Software, release 13.0, StataCorp, College Station, TX, USA) to examine the data with regard to group codification and allocation.

## 3. Results

**Figure 5:**
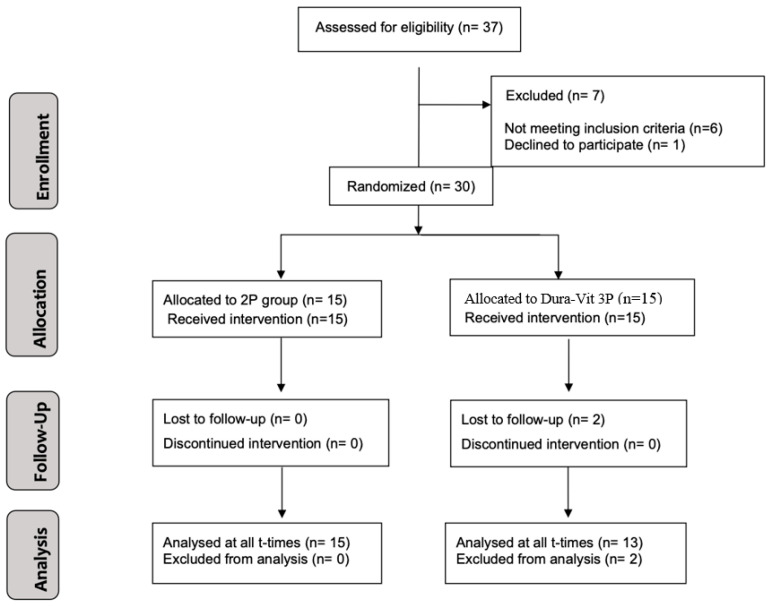
Flow diagram illustrating the progression of study participants according to CONSORT guidelines.

The CONSORT diagram illustrating the subject flow throughout the trial is presented in Figure 5. A total of 30 patients (19 females and 11 males) were enrolled and treated between September 2020 and June 2021. Two patients from the Dura-Vit 3P group dropped out after implant placement, resulting in the absence of follow-up CBCT data. One relocated and continued prosthetic rehabilitation at another dental center, which confirmed the implant’s successful survival, while the other patient could not be contacted. The average age of participants was 52.7 years (SD 19.5 years).

Table 1 summarizes the demographic and surgical site characteristics for the two treatment groups considering all patients that received implant placement. There was a quite homogenous distribution of sex, smoking habits, and gingiva biotype between the two groups.

An intraclass correlation coefficient value of 0.92 (mean error 0.11 mm) indicated satisfactory agreement with intra-examiner repeatability for CBCT measurements.

Table 2 reports implant characteristics, position and insertion torque for each group. Regarding the position, the dental implants placed were divided into three groups: the molar region, comprising the first and second molars; the premolar region, comprising the first and second premolars; and the anterior region, comprising the incisors and canines.

The distribution of implants across these groups was approximately similar. A significant difference was found in the insertion torque, with the Dura-Vit 3P group exhibiting a mean of 5.2 Ncm lower than the System 2P group (*p*-value = 0.005). One implant of the System 2P group was lost 3 months after insertion. The implant exhibited mobility and was removed easily from the implantation site. At the 6-month follow-up and re-opening, no additional indications of peri-implant inflammation or problems were seen for any of the remaining implants.

Implant stability quotients (ISQs) at T1 and T2 for each group and jaw are reported in Table 3. As a general tendency, the mean ISQ was higher for the mandible than for the maxilla and increased at T2 compared to T1.

A significant difference between groups was observed at T1, with the Dura-Vit 3P resulting in higher ISQs for both the maxillary implants (*p* value = 0.001) and the mandibular ones (*p*-value = 0.001). No substantial difference between groups was observed at T2 for any of the jaws (*p* < 0.05).

Table 4 shows the variations in vertical gap and horizontal bone diameters at T1 and T2 within and between groups.

At baseline, no relevant difference between groups was assessed for any of the outcomes. Comparison between T2 and T1 showed that mean horizontal resorption occurred in both groups and the most significant changes occurred at the bevel/marginal level on both the vestibular and palatal side. There was some minimal bone gain for the Dura-Vit 3P group at the medial and apical level of the palatal side. Significant differences were seen at the vestibular and palatal base and medial level (*p*-values < 0.05), with the Dura-Vit 3P group showing a lesser reduction than the System 2P for all other outcomes.

Regression models, adjusted for implant type, surgeon, and baseline values, indicated that a thin gingival biotype was associated with reduced bone loss at the vestibular base level (0.1, *p* = 0.025). The smoker status did not show a predictive effect on the horizontal bone changes at the base level in either the vestibular or the palatal side (0.73, *p* = 0.18 and 0.39, *p* = 0.22, respectively). The implant diameter and the length did not influence the ISQ (0.32, *p* = 0.12, and 0.12, *p* = 0.31, respectively) or the crestal bone changes (*p*-value < 0.05 for both variables at apical, medial, marginal, and vertical measurements). No difference was observed between the three surgeons for any of the outcomes.

The vertical gap was reduced for all of the implants. No significant difference between groups was observed at both T1 and T2.

The thin gingival biotype had no influence on lowering the vertical gap, according to regression models that controlled for baseline values, surgeon type, and implant type (−0.9, *p* = 0.07). Likewise, the decrease in the vertical gap was unaffected by smoking or surgery (0.48, *p* = 0.12 and 0.13, *p* = 0.24, respectively).

## 4. Discussion

Dental implant macro-geometry is one major variable that influences osseointegration biology and biomechanics and therefore directly affects the success of the implants [26]. In clinical settings where achieving primary stability is difficult, including post-extraction sites or cases with poor bone density, the design elements are very crucial.

In the present study, we investigated the influence of implant macro-design on primary stability and peri-implant bone behavior by comparing two implants of different macro-designs inserted in post-extraction sites. Both clinical and radiographic parameters were measured.

The surgical technique was standardized and excluded flap opening, bone grafting, or other types of peri-implant management that might influence osseointegration and bone response to avoid clinical biases and to allow for a straightforward comparison of implants. Furthermore, based on our previous research and in agreement with the current scientific evidence, the advantages of bone grafting on osseointegration of post-extraction implants are questionable. Therefore, it was considered of no benefit for the patients and not implemented in any of the cases.

The System 2P implant exhibited higher insertion torque as was expected due to increased interface contact area with the surrounding bone walls and therefore higher percentage of bone-to-implant contact (BIC) [27,28]. Despite this, the Dura-Vit 3P implant showed higher ISQ scores in both the maxillary and mandibular arch, indicating greater primary stability. Similarly, the Dura-Vit 3P implant resulted in significantly better buccal bone preservation, especially at the medial and marginal levels. Considering that both implants have the same surface treatment, the differences in geometry features might be the main factor explaining these results.

The different pitch, thread depth, thread distribution, and angles are important aspects that can influence primary stability, subsequent osseointegration, and bone remodeling [17].

The Dura-Vit 3P implant macro-geometry is developed with larger thread depth and pitch, allowing for increased healing chambers and low-compression areas of bone tissue during implant insertion and stabilization that might accelerate and improve osseointegration. As previously demonstrated in pre-clinical and clinical studies, the healing chamber design allows for blood clot apposition and a higher presence of osteocytes during both implant placement and bone healing [29]. Furthermore, it is demonstrated that deeper threads have an important effect on implant stabilization, especially in clinical situations representing poorer bone quality [20].

As for the shape, taking as reference the external diameter of the threads, the Dura-Vit 3P implant tends to have a more tapered shape compared to the System 2P implant. It is demonstrated that this shape exhibits better stress distribution and primary stability than cylindrical-shaped implants. In the case of the System 2P implants, the more cylindrical shape, associated with higher insertion torque and bone compression, might cause increased bone resorption and peri-implant bone loss [30]. The more tapered shape also contributes to a reduced likelihood of perforation in the buccal bone plate, but particular attention should be paid to choosing the correct diameter, especially at the medial level of the implant [17]. Our results did not promote the use of a specific diameter for any of the implants. This was to be expected as the post-extractive sites of different teeth have unpredictable shapes and the primary stability is mainly assured by engagement at the apical level. Therefore, the appropriate diameter was chosen based on the shape and diameter of the post-extraction site and available bone. For the Dura-Vit 3P implant, the apical face angle (internal angle) is higher, and this promotes the generation of shear forces at the bone–implant apical interface that facilitates the threading. This characteristic is particularly beneficial in post-extraction implants engaging mainly at the available apical bone.

Another feature that can translate into differences in stability and peri-implant tissue reaction is represented by neck structure and height. It has been shown that the micro-rings on the implant neck might increase stability and reduce crestal bone loss [31,32]. The Dura-Vit 3P implant incorporates different thread pitches and shapes along the implant body length. Based on the current scientific evidence, the manufacturer has incorporated shallower and closer threads at the neck level. This feature promotes the preservation of marginal bone, while the more apical wide threads stabilize the bucco-lingual bone diameter.

In terms of bone type and jaw, we did not observe important differences in implant performance between the upper and lower jaw. This suggests that the Dura-Vit 3P implant is appropriate and promotes bone preservation in post-extraction sites not only in the less dense bone like the one in the maxilla but also in high-density bone such as the mandible.

In the present study, implants were inserted by three different experienced surgeons following the same protocol as suggested by the manufacturer. No differences between surgeons were observed for any of the outcomes even though all of them had no experience with the Dura-Vit 3P implant. These findings are in agreement with previous studies reporting that the influence of the surgical technique is smaller than that of implant size and shape [26,33].

It is interesting to note that smoker status did not demonstrate a predictive effect on horizontal bone changes at the base level. This finding is somewhat unexpected, given that smoking is typically associated with adverse effects on bone health and remodeling [34], potentially even leading to implant failure [35]. However, this finding may be explained by the study’s inclusion criteria, which limited participants to those smoking fewer than 10 cigarettes per day, whereas most studies evaluating the impact of smoking on the periodontium define smokers as individuals who smoke more than 10 cigarettes per day [36]. Such a low level of tobacco exposure may not have been sufficient to induce significant alterations in bone dynamics, thereby mitigating the expected impact of smoking on horizontal bone changes.

In terms of limitations, external validity should be considered. Our results can be extended to the majority of post-extraction implant cases as implants were inserted in different teeth in both the upper and lower jaw. However, some inclusion and exclusion criteria do not allow for the transfer of findings to all post-extraction everyday cases. Also, implants were inserted by experienced surgeons with extensive clinical experience in post-extraction implants, which might influence the clinical achievements [35]. Furthermore, the present study investigated the first 6-month healing phase with no prosthetic loading, aiming to determine primary stability and bone reaction. However, with respect to the System 2P implant, the design of the Dura-Vit 3P implant has some differences: (1) the incorporation of a micro-threaded collar that helps to increase primary stability (the System 2P implant has a transmucosal collar) and soft tissue healing and reduce vertical prosthesis load, (2) the combination of a triple-thread spiral with 60° beveled profile threading, which increases the mating surface with the bone to improve osseointegration, and (3) the presence of an apical rounded shape to reduce the risk of perforation, especially in the maxillary area, which could be appropriate not only for immediate implantation but for immediate loading as well [37]. This might represent a further advantage for clinical indications that should be tested in future studies. Another limitation to take into account is the inherent nature of radiographic examinations, as the absence of a gap detected through CBCT does not necessarily confirm proper osseointegration or the quality of the newly formed tissue on the implant surface. Lastly, although the overall sample size met statistical requirements, we did not perform further subdivision into subgroups (molar, premolar, anterior regions, and different implant designs) as this would have reduced statistical power, limiting the ability to detect significant differences. This study provides critical insights into the impact of implant macro-design on primary stability and bone remodeling. The findings underscore the Dura-Vit 3P implant’s superior primary stability and enhanced preservation of crestal bone in post-extraction sites, attributed to its innovative macro-design, including apical self-tapping features and larger thread depth. These results significantly advance the understanding of implant geometry’s role in osseointegration and offer valuable clinical recommendations for post-extraction implantology. Further investigations with a larger patient cohort are needed to analyze additional variables, such as implant position, bone quality, and surgical technique, which may influence the relationship between implant geometry and bone remodeling. This stratified approach will refine clinical strategies and further improve outcomes.

### Strong Points and Novelty

This study stands out due to its rigorous randomized controlled trial (RCT) design, standardized surgical procedures, and the use of CBCT for precise measurement of peri-implant bone changes. The novel application of an innovative macro-design in the Dura-Vit 3P implant demonstrates a unique combination of features, such as apical self-tapping and increased thread depth, which significantly enhance primary stability and promote crestal bone preservation. These findings not only establish a direct link between implant geometry and improved clinical outcomes in post-extraction sites but also highlight the potential for these implants to redefine approaches in implantology. Furthermore, this study provides one of the first pieces of evidence suggesting that specific macro-design elements can mitigate bone loss and optimize osseointegration, laying the groundwork for future investigations into implant design and immediate prosthetic loading.

## 5. Conclusions

In conclusion, the findings of this randomized clinical trial indicate that no flap–no graft surgery for post-extraction implants, when adequate buccal bone is available, offers a minimally invasive and well-tolerated approach that achieves satisfactory outcomes in terms of ‘jump space’ filling and dimensional bone preservation. Furthermore, as the study is based on a short 6-month observation period, longer-term follow-up is necessary to further confirm the stability of the facial bone and soft tissues over time.

## Figures and Tables

**Table 1:** Patient characteristics by treatment groups.

Patient Data	Treatment Group	
	System 2P	Dura-Vit 3P
Age (mean, SD)	50.3 (15.8)	55.1 (12.7)
Sex		
Female	8	11
Male	7	4
Smoke		
Yes (1–10/day)	4	3
No	11	12
Gingiva biotype		
Thin	5	7
Thick	10	8

**Table 2:** Implant characteristics by treatment groups.

	Jaw						Diameter		Insertion Torque
	Upper			Lower			3.5 mm	4 mm	Mean (SD)
	Molar	Premolar	Frontal	Molar	Premolar	Frontal			
System 2P	4	4	1	3	2	1	4	11	44.5 (5.4)
Dura-Vit 3P	4	2	1	4	3	2	12	3	49.7 (2.9)

**Table 3:** Implant stability quotient (ISQ) measured for each group at T1 and T2, reported by jaw.

		T0 Mean (SD)	T1 Mean (SD)
System 2P	Maxilla	51.25 (4.39)	67.32 (5.24)
	Mandible	56.83 (6.75)	75.89 (7.21)
Dura-Vit 3P	Maxilla	68.94 (7.12)	70.16 (5.38)
	Mandible	77.98 (5.74)	79.47 (6.82)

**Table 4:** Horizontal and vertical bone level changes. Mean (mm) and standard deviations (SDs) are reported for each variable at T1 and T2 and for the difference T2-T1. The *p*-value of Wilcoxon tests for pairwise comparisons between groups is reported in the last column. *: *p* < 0.05.

	System 2P Mean (SD)	Dura-Vit 3P Mean (SD)	*p*-Value
**Palatal/lingual base**			
T1	2.77 (1.97)	2.25 (1.21)	0.421
T2	2.21 (1.85)	2.19 (1.01)	0.972
T2-T1	−0.56 (0.62)	−0.06 (0.46)	0.026 *
**Palatal/lingual medial**			
T1	3.08 (2.46)	2.59 (1.84)	0.558
T2	2.93 (2.44)	2.96 (1.51)	0.643
T2-T1	−0.15 (0.66)	0.37 (0.41)	0.022 *
**Palatal/lingual apex**			
T1	4.07 (2.65)	4.25 (2.16)	0.846
T2	4.01 (2.60)	4.47 (2.06)	0.531
T2-T1	−0.06 (0.55)	0.22 (0.26)	0.108
**Vestibular base**			
T1	1.57 (1.27)	1.46 (1.41)	0.833
T2	1.02 (1.04)	1.43 (1.14)	0.348
T2-T1	−0.55 (0.48)	−0.03 (0.56)	0.016 *
**Vestibular medial**			
T1	3.74 (1.16)	3.01 (1.28)	0.133
T2	3.02 (1.46)	2.98 (1.08)	0.937
T2-T1	−0.72 (0.70)	−0.02 (0.92)	0.035 *
**Vestibular apex**			
T1	5.32 (2.93)	4.87 (2.62)	0.679
T2	5.07 (1.32)	4.83 (1.44)	0.655
T2-T1	−0.25 (0.53)	−0.04 (0.83)	0.437
**Vertical gap gap**			
T1	6.12 (3.21)	5.09 (3.82)	0.454
T2	2.25 (1.59)	1.02 (1.47)	0.132
T2-T1	−3.87 (0.88)	−4.07 (0.19)	0.431

## Data Availability

The data to support the findings of this study will be available on request from the corresponding author.
